# Reported human infections of H9N2 avian influenza virus in China in 2021

**DOI:** 10.3389/fpubh.2023.1255969

**Published:** 2023-12-08

**Authors:** Min Tan, Xiaoxu Zeng, Yiran Xie, Xiyan Li, Jia Liu, Jiaying Yang, Lei Yang, Dayan Wang

**Affiliations:** ^1^National Institute for Viral Disease Control and Prevention, Chinese Center for Disease Control and Prevention, National Key Laboratory of Intelligent Tracking and Forecasting for Infectious Disease, Beijing, China; ^2^School of Public Health (Shenzhen), Sun Yat-sen University, Guangdong, China

**Keywords:** H9N2, avian influenza virus, poultry, zoonotic, human infections

## Abstract

**Introduction:**

The continued emergence of human infections of H9N2 avian influenza virus (AIV) poses a serious threat to public health. The prevalent Y280/G9 lineage of H9N2 AIV in Chinese poultry can directly bind to human receptors, increasing the risk of spillover infections to humans. Since 2013, the number of human cases of H9N2 avian influenza has been increasing continuously, and in 2021, China reported the highest number of human cases, at 25.

**Methods:**

In this study, we analyzed the age, geographic, temporal, and sex distributions of humans with H9N2 avian influenza in 2021 using data from the National Influenza Center (Beijing, China). We also conducted evolutionary, gene homology, and molecular characterization analyses of the H9N2 AIVs infecting humans.

**Results:**

Our findings show that children under the age of 12 accounted for 80% of human cases in 2021, and females were more frequently affected than males. More cases occurred in winter than in summer, and most cases were concentrated in southern China. Human-infecting H9N2 viruses showed a high level of genetic homology and belonged to the prevalent G57 genotype. Several additional α2,6-SA-binding sites and sites of mammalian adaptation were also identified in the genomes of human-infecting H9N2 viruses.

**Discussion:**

Therefore, continuous monitoring of H9N2 AIV and the implementation of further measures to control the H9N2 virus in poultry are essential to reduce the interspecies transmission of the virus.

## Introduction

Since the 1990s, the H9N2 avian influenza virus (AIV) has been detected in poultry and mammalian species, including chickens, ducks, smaller poultry, and pigs, in China (1–3). The virus has continuously evolved, resulting in multiple lineages, of which the G1, G9/Y280, and Y439 lineages continue to circulate in poultry (4, 5). The G9/Y280 lineage of H9N2 AIV has predominated in China in recent years. Since the emergence of the G57 genotype, which is better adapted to chickens, it has become predominant and has caused widespread outbreaks (1, 6).

Ongoing epidemics of H9N2 AIV in poultry increase the risk of spillover infections in humans. The first case of human H9N2 AIV infection was detected in Hong Kong SAR, China, in 1998. Since 2013, there have been increasing reports of human infections of H9N2 AIV (7). Several studies have shown that the majority of recent avian-origin H9N2 AIVs have a strong binding affinity for a human respiratory receptor (7, 8), and serological surveys have shown higher positivity among poultry-associated workers (9, 10). Therefore, assessing the pandemic risk of current H9N2 AIV genotypes is important and urgent.

In this study, we collected and analyzed data on human H9N2 AIV infections in China in 2021. We examined the epidemiological and genetic characteristics of the virus to extend our understanding of human infections and the risks they pose.

## Methods

### Research background

In China, all laboratory-diagnosed cases of H9N2 influenza in 2021 were reported through the national surveillance system. Patients whose respiratory specimens tested positive for H9 and N2 with real-time reverse transcription (RT)–PCR were confirmed as infected with H9N2. Demographic and epidemiological data on these cases of H9N2 influenza were collected with standard forms, including information on age, sex, and place of residence.

### Ethical considerations

The National Health Commission of the People’s Republic of China has deemed that collecting data on each H9N2-infected patient is part of ongoing public health investigations into emerging infectious diseases. Therefore, our Institutional Review Board waived the requirement for formal ethical approval.

### Virus isolation and sequencing

Original samples from human patients were collected for an H9N2 subtype analysis with real-time RT–PCR by the local CDCs (Ct less than 38 were considered positive, while those with Ct values between 38 and 40 were subjected to repeat experiments due to the requirement for confirmation. Samples with Ct values greater than 40 were regarded as negative). The H9N2 virus was isolated from positive samples in a biosafety level 2 laboratory. Nine-day-old specific-pathogen-free embryonated chicken eggs were inoculated with an aliquot (0.2 mL) of the original sample. After incubation at 37°C for 48 h, we harvested the egg allantoic fluid, extracted the RNA from it, and sequenced the genome original samples from human patients and viral samples (11).

### Virus sequencing

We used the Illumina next-generation sequencing (NGS) technology on the MiSeq platform (Illumina, Inc., San Diego, CA, United States) to sequence the human H9N2 influenza virus. Following the acquisition of the viral sequences, we conducted a series of analysis steps. First, we checked the quality of the sequences, excluding any potential sequencing errors or contamination. We then assembled high-quality reads using bioinformatic tools like Velvet (version 1.2.10) and Newbler (version 2.5) to align the viral sequences with known reference sequences of H9N2 viruses in the Global Initiative on Sharing All Influenza Data (GISAID).^1^ This involved detecting single-nucleotide variations (SNVs), insertion/deletion of variants, and identifying variable sites that could potentially lead to changes in protein sequences. Bowtie 2 (version 2.1.0) was used to map the reads using sequences with the maximum similarity.

### Sequence alignment and phylogenetic analysis

We obtained sequences from 16 human isolates and downloaded H9N2 AIV reference sequences from GISAID: 37 sequences of polymerase basic protein 1 (PB1), 34 of polymerase basic protein 2 (PB2), 34 of neuraminidase (NA), 55 of hemagglutinin (HA), 35 of matrix protein (M), 37 of nucleoprotein (NP), 38 of non-structural protein (NS), and 35 of polymerase (PA). We constructed maximum likelihood phylogenetic trees for each gene segment of the selected influenza viruses with the GTR model in MEGA 7.0, with 1,000 bootstrap replicates.

## Results

### Epidemiological characteristics of H9N2 AIV infections in humans

**Figure 1:**
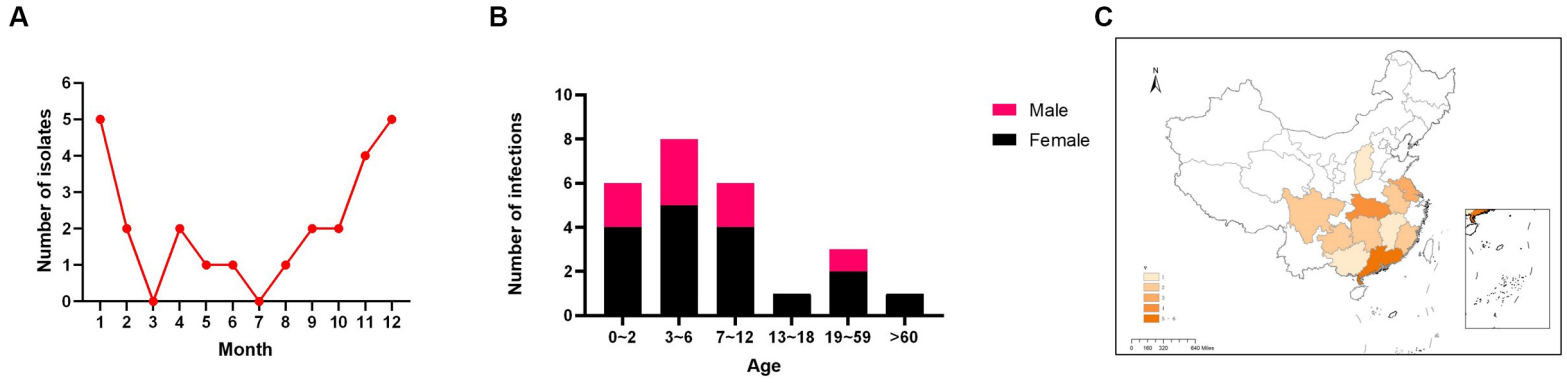
Distribution of cumulative reported cases of human H9N2 avian influenza virus infection in China from January to December 2021 **(A)**. Age and sex distributions of cases of human H9N2 avian influenza in 2021. Black, female; Red, male **(B)**. Regional distribution of human cases of H9N2 avian influenza virus in China in 2021. The darker the color, the greater the number of infections **(C)**.

The first reported case of human infection with H9N2 AIV was in China in 1998 (12), and since then, continuously increasing numbers of human infections have been recorded. In 2021, China reported a record number of 25 cases of human H9N2 AIV infection. All reported patients presented with flu-like symptoms, except patients who died, in whom the infection was associated with underlying health conditions. A seasonal pattern was observed, with more cases occurring in winter than in summer. Specifically, 16 of 25 cases (64%) occurred in January, February, November, and December 2021 (Figure 1A).

The 25 infections were predominantly in children. The median patient age was 5 years (interquartile range: 2.5–9.5). The subdivided age groups showed that among the infected individuals, six (24%) were infants aged 1–2 years, eight (32%) were children aged 3–6 years, six (24%) were children aged 7–12 years, one (4%) was a teenager aged 13–18 years, three (12%) were adults, and one (4%) was an older adult (Figure 1B). These data indicate that the majority of H9N2 AIV infections occurred in children under 12 years old, who accounted for 80% (20/25) of cases. Of all the patients, 8 were male and 17 female subjects, resulting in a male-to-female ratio of 1:2.125.

These patients came from 11 provinces, municipalities, or autonomous regions (Guangdong, Hubei, Jiangsu, Guangxi, Guizhou, Fujian, Sichuan, Anhui, Jiangxi, Hunan, and Shanxi), and most were from Southern China (96%, 24/25 patients). Guangdong Province had the highest number of cases, with six patients (Figure 1C).

### Genetic characteristics of H9N2 AIV isolated from humans

**Figure 2:**
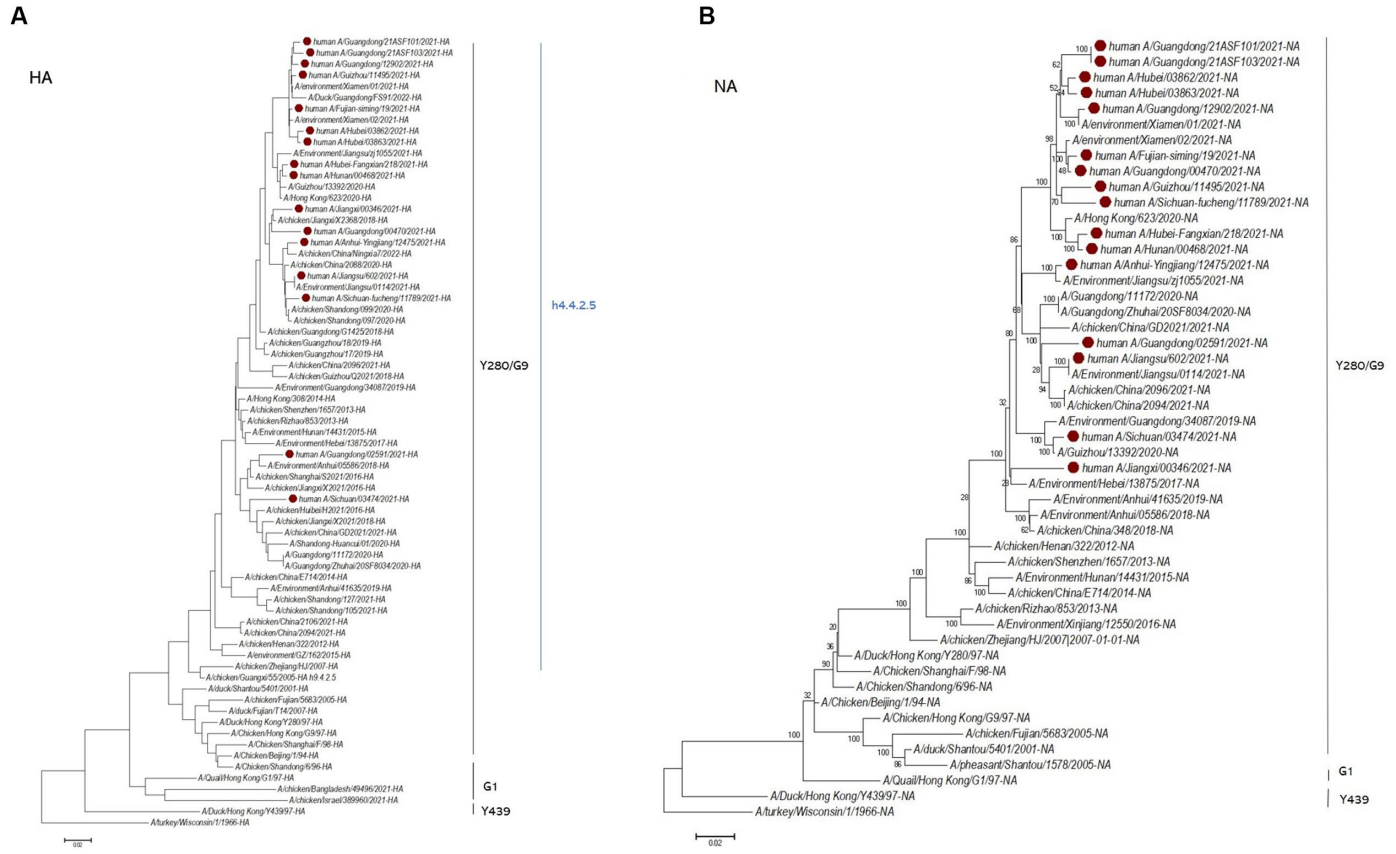
Maximum likelihood phylogenetic tree of the HA gene **(A)** and NA gene **(B)**. The H9N2 virus infections in Chinese people in 2021 analyzed in this study are marked in red.

**Figure 3:**
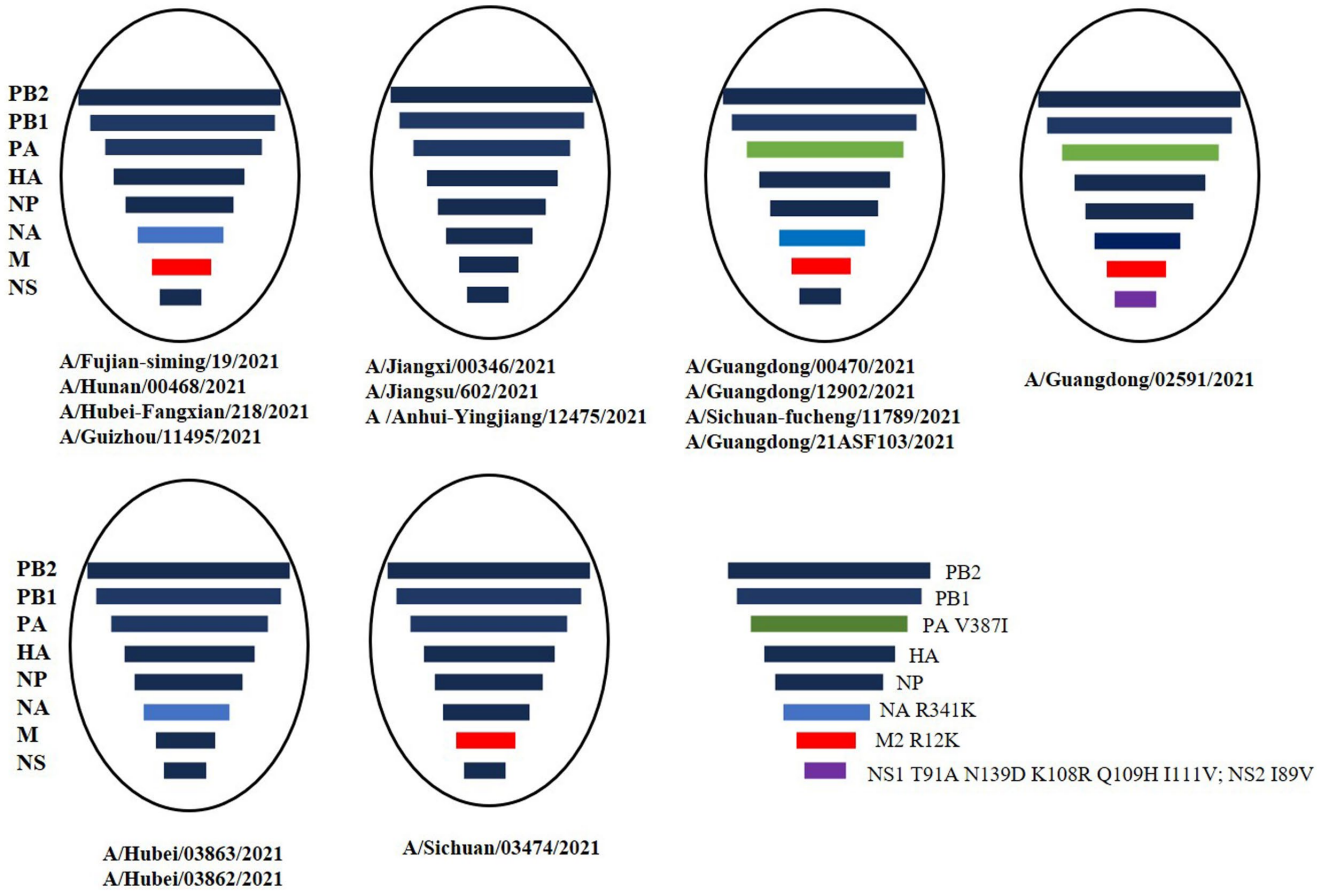
The 16 human H9N2 viruses. The eight gene segments of the H9N2 viruses, represented by horizontal bars are, from top to bottom, PB2, PB1, PA, HA, NP, NA, M, and NS. Each different color represents a distinct branch.

A total of 16 viruses were isolated from 25 patients. Phylogenetic analysis of the HA gene showed that they all belonged to the G9/Y280 lineage. The NA and internal genes of the human isolates were located in the same clades as those of the representative strain of the G57 genotype, A/chicken/Zhejiang/HJ/2007 (Figure 2; Supplementary Figures S1–S6). These human isolates showed high genetic diversity and shared 91.8–99.2% nucleotide homology in HA, 92.8–99.8% in NA, 93.4–99.7% in PB2, 92.1–99.8% in PB1, 93.9–99.5% in PA, 94.8–99.5% in NP, 95.1–99.8% in MP, and 93.9–99.5% in NS. We also categorized the 16 human H9N2 strains sequences into six subgenotypes (Figure 3). The eight gene segments of the H9N2 viruses, represented by horizontal bars are, from top to bottom, PB2, PB1, PA, HA, NP, NA, M, and NS. Each color represents a distinct origin.

All 16 human viruses had leucine at position 226 (H3 numbering) of HA, which may increase the virus’s binding affinity for α-2,6 sialic acid (α2,6-SA), the human AIV receptor. Other mutations, such as 155 T and 190 T/V (13), which may also increase viral binding to the human receptor, were also found in some of the isolates (Table 1; Supplementary Figures S7, S8).

**Table 1:** Inventory of major molecular markers affecting biological characteristics of avian influenza A viruses.

Protein	Function	Mutations	Amino acids	Human viruses	References
PB2	Increased polymerase activity, increased viral replication, and virulence in mice	I292V	I	5	(14)
			V	11	
		A588V	V	16	(15)
		E627K	E	10	(16)
			K/E	1	
			V	5	
		D701N	D	16	(17)
	Increased human infection	K702R	K	7	(18)
			R	9	
	Increased mammalian adaptation	A558V	T	16	(15)
		D253N	D	16	
		K340R	R	6	(15)
			K	10	
		K526R	D	16	(19)
	Increased replication in mammalian cell line, increased virulence in mice	Q591K	Q	16	(20)
PB1	Increased ferret infection	I368V	V	15	(21)
			I	1	
	Increased mammalian adaptation	D120T	D	16	(22)
		D439Y	D	16	(23)
		S261Y	R	1	(24)
			S	15	
PA	Increased mammalian replication and pathogenicity	356R	R	16	(25)
	Increased human infection	409 N	N	15	(26)
			S	1	
	Increased replication and higher morbidity and mortality rates along with extended tissue tropism seen in chickens.	K26E	E	16	(27)
HA*	Increased virus binding to α2’6-SA	155 T	T	2	(28)
			N	13	
			Y	1	
		183 N	N	16	(28)
		226 L	L	16	(29–31)
	Enhanced virus binding to α2’6-SA and increased replication and virulence in mice	190 T/V	V	10	(13)
			T	6	
HA2	Increase airborne transmission	G192R	R	16	(32–34)
M2	Increased resistance to amantadine and rimantadine	S31N/G	N	15	(35)

*H3 numbering system was used.

Residues previously identified as enhancing the polymerase activity, virulence, and transmission of AIVs were examined. One virus had PB2 627 K/E, whereas five had 627 V, which may confer a virulent phenotype on H9N2 AIV in mice (36). Eleven viruses had PB2 292 V and all 16 had PB2 588 V, which are markers of mammalian adaptation and enhance the viral polymerase activity, replication, and virulence in mammals (14, 15). Nine viruses in this study had PB2 702R, which is a species-specific residue of human influenza viruses (37).

We examined mutation sites associated with reduced drug susceptibility and identified M2-S31N in 16 viral strains, which confers resistance to adamantanes (38). No drug resistance mutations were found for NA inhibitors or PA inhibitors, such as NA-136 K, NA-292 K, PA-38 M/T, or PA-37 T.

## Discussion

In this study, we analyzed the human H9N2 AIV infections reported in China in 2021, the year in which the highest number of H9N2 cases was recorded to date. We examined the epidemiological and genetic characteristics of the patients and isolates. H9N2 infections occurred most frequently in winter in Southern China and mainly occurred in children under 12 years old, who accounted for 80% of all cases. More female subjects were infected than male subjects. Therefore, the prevalence of H9N2 AIVs must be closely monitored to prevent cross-species transmission.

Host barriers largely restrict the cross-species transmission of AIVs. In recent years, the α2,6-SA-binding ability of H9N2 has increased continuously, and the predominant strains that have emerged show dual receptor binding or preferentially bind α2,6-SA (8, 39). HA Q226 L plays a critical role in receptor binding and was detected in all 16 viruses in this study. Moreover, increased α2,6-SA-binding sites, such as 155 T, 183 N, and 190 V, were also found in the H9N2 viruses that infected humans. Although most H9N2 viruses do not have well-known mammalian adaptation markers, such as PB2 627 K and 701 N, several amino acid residues that facilitate mammalian adaptation were detected, including PB2-588 V, PB1-368 V, PA-356R, and PA-409 N.

Throughout its evolution, the genetic diversity of H9N2 AIV has increased, with mutations that adapt the virus to mammalian hosts and may increase the risk of human infection. High levels of H9N2 AIV among birds, especially poultry, may also increase the opportunities for virus spillover and human infections. Serious efforts are required to constrain the virus in poultry. Continued monitoring of H9N2 AIV, including its close genetic surveillance and phenotypic characterization in animal models, should be incorporated into risk assessment strategies.

## Conclusion

Since 2013, the number of human cases of H9N2 avian influenza has been steadily increasing, and in 2021, China reported the highest number of human cases, at 25. Children under the age of 12 accounted for 80% of human cases in 2021, and female subjects were more frequently affected than male subjects. More cases occurred in winter than in summer, and most cases were concentrated in Southern China. Human-infecting H9N2 viruses showed a high level of genetic homology and belonged to the prevalent G57 genotype. Continuous monitoring of H9N2 avian influenza viruses and further measures to control the H9N2 virus in poultry are essential to reduce the interspecies transmission of the virus.

## Data availability statement

The datasets presented in this study can be found in online repositories. The names of the repository/repositories and accession number(s) can be found in the article/Supplementary material.

## Author contributions

MT: Data curation, Visualization, Writing – original draft, Methodology. XZ: Data curation, Methodology, Writing – review & editing. YX: Investigation, Writing – review & editing. XL: Data curation, Methodology, Writing – review & editing. JL: Methodology, Writing – review & editing. JY: Methodology, Writing – review & editing. LY: Validation, Writing – review & editing. DW: Funding acquisition, Writing – review & editing, Supervision.

## Footnotes

^1^http://www.gisaid.org
