# Supplementary figures and images for: Reported human infections of H9N2 avian influenza virus in China in 2021
Source: Front Public Health. 2023 Dec 8;11:1255969. doi: 10.3389/fpubh.2023.1255969 (PMC10753182; doi:10.3389/fpubh.2023.1255969)

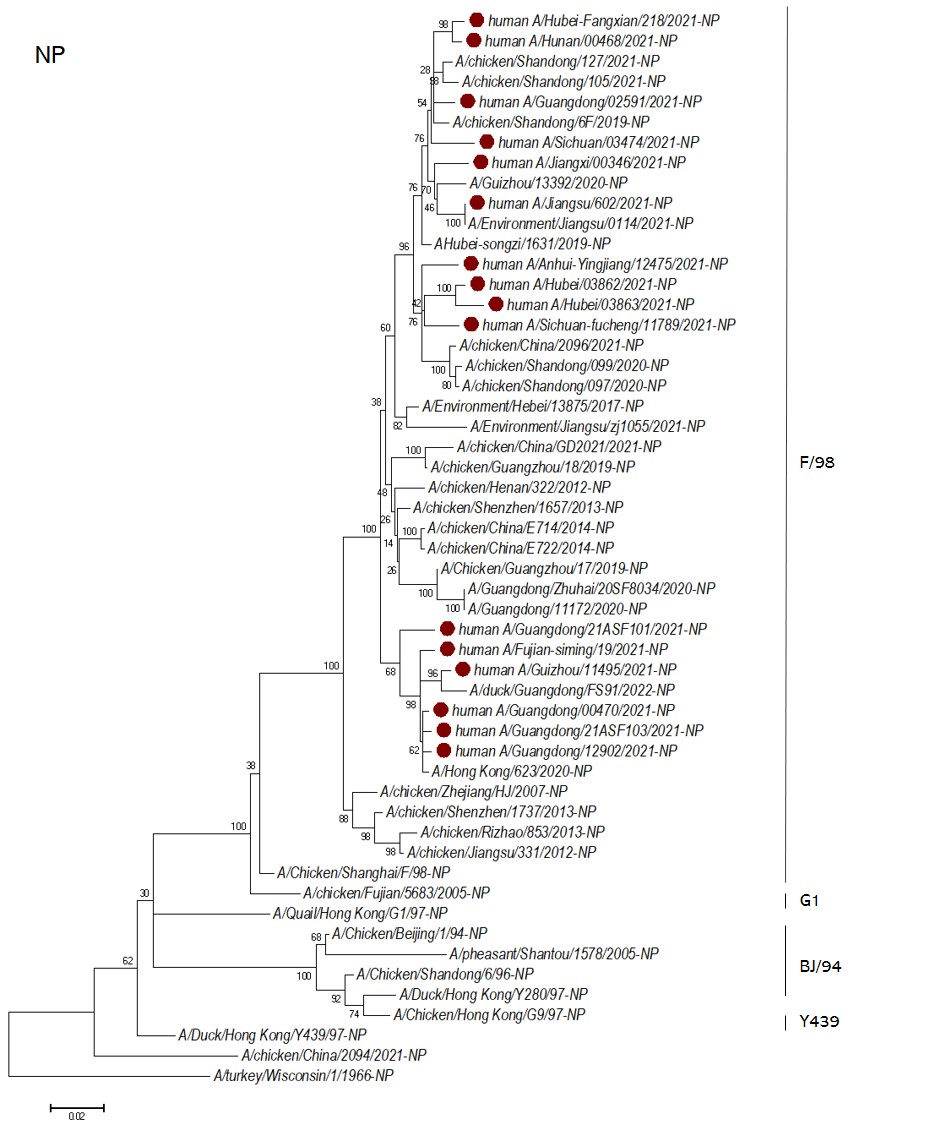

Supplement: Supplementary file 1 [file Image_1.JPEG]

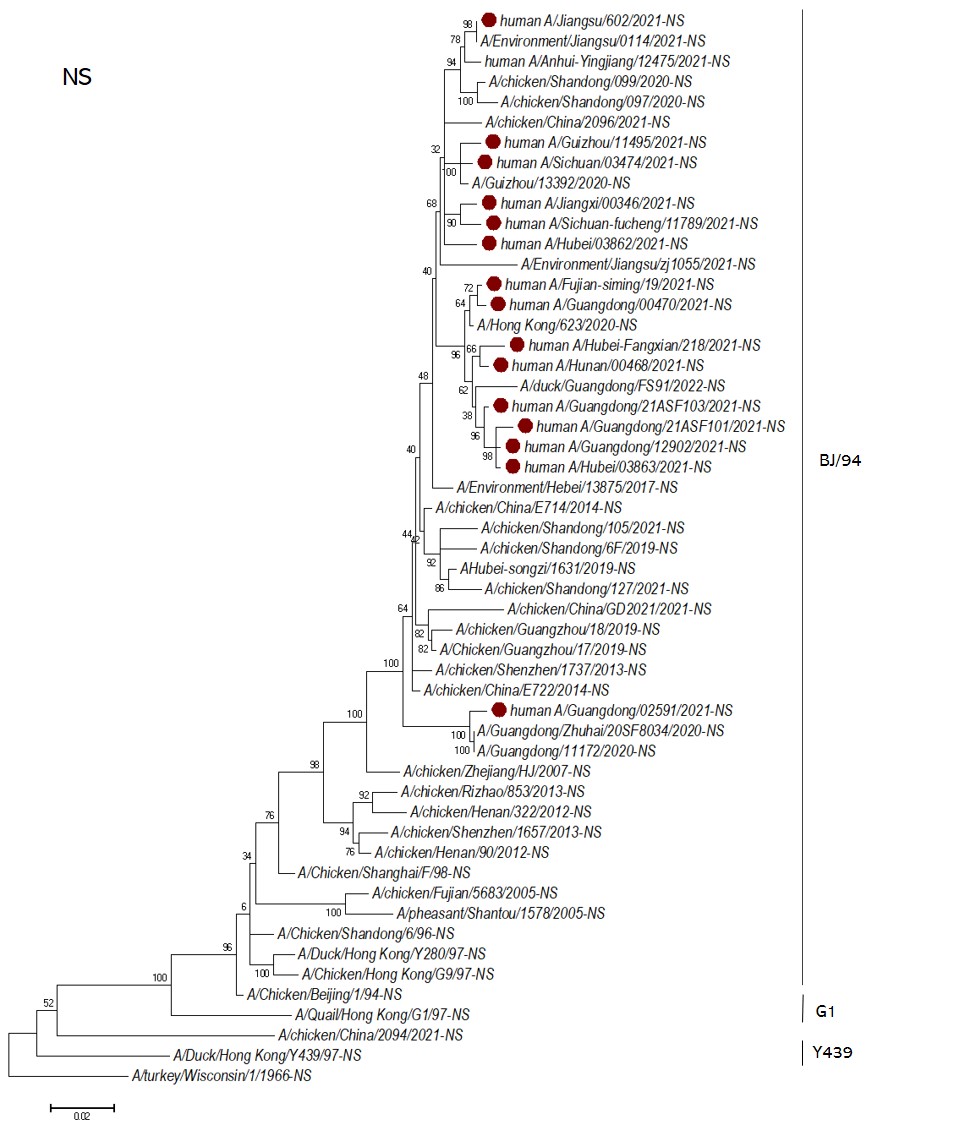

Supplement: Supplementary file 2 [file Image_2.JPEG]

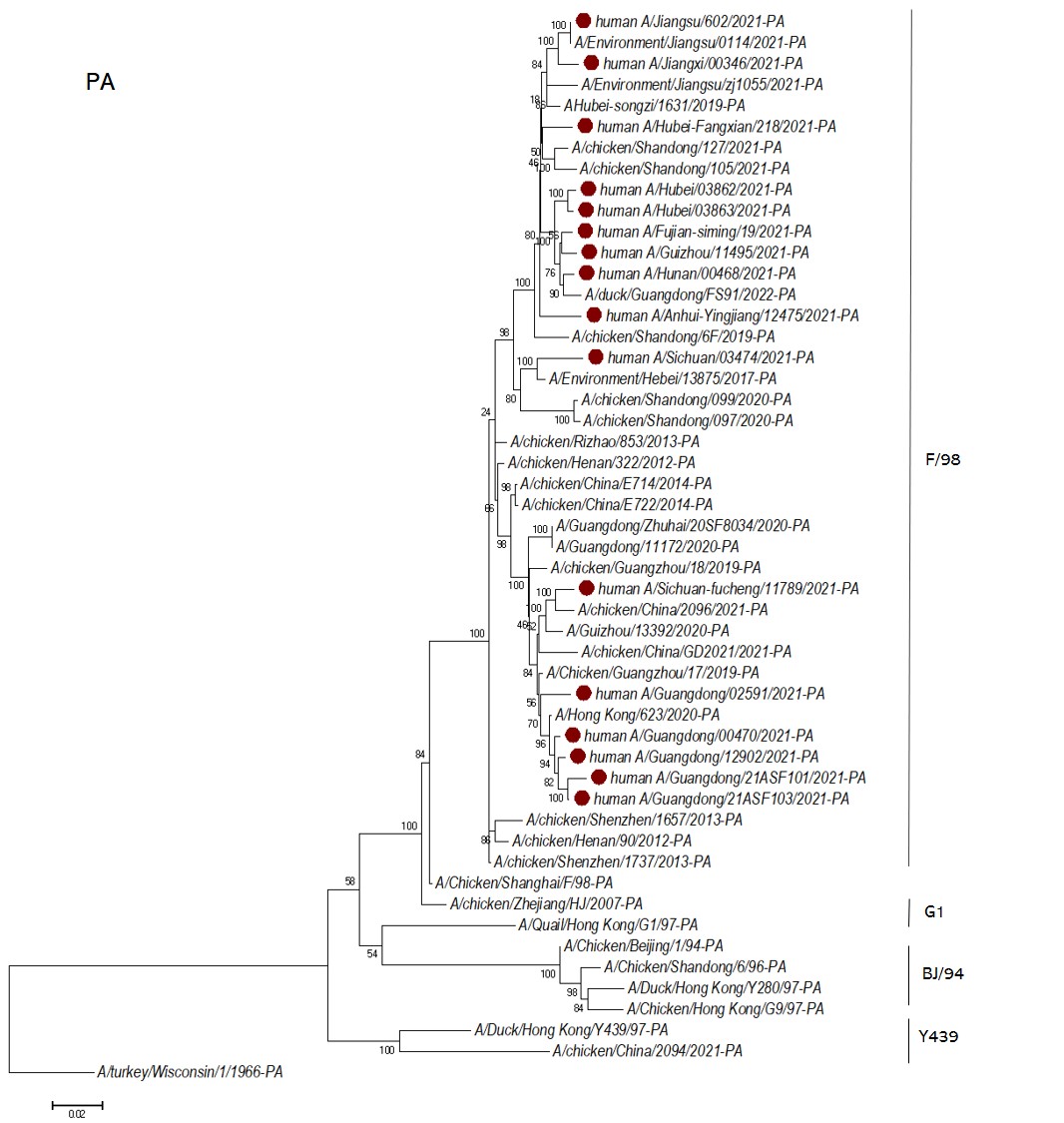

Supplement: Supplementary file 3 [file Image_3.JPEG]

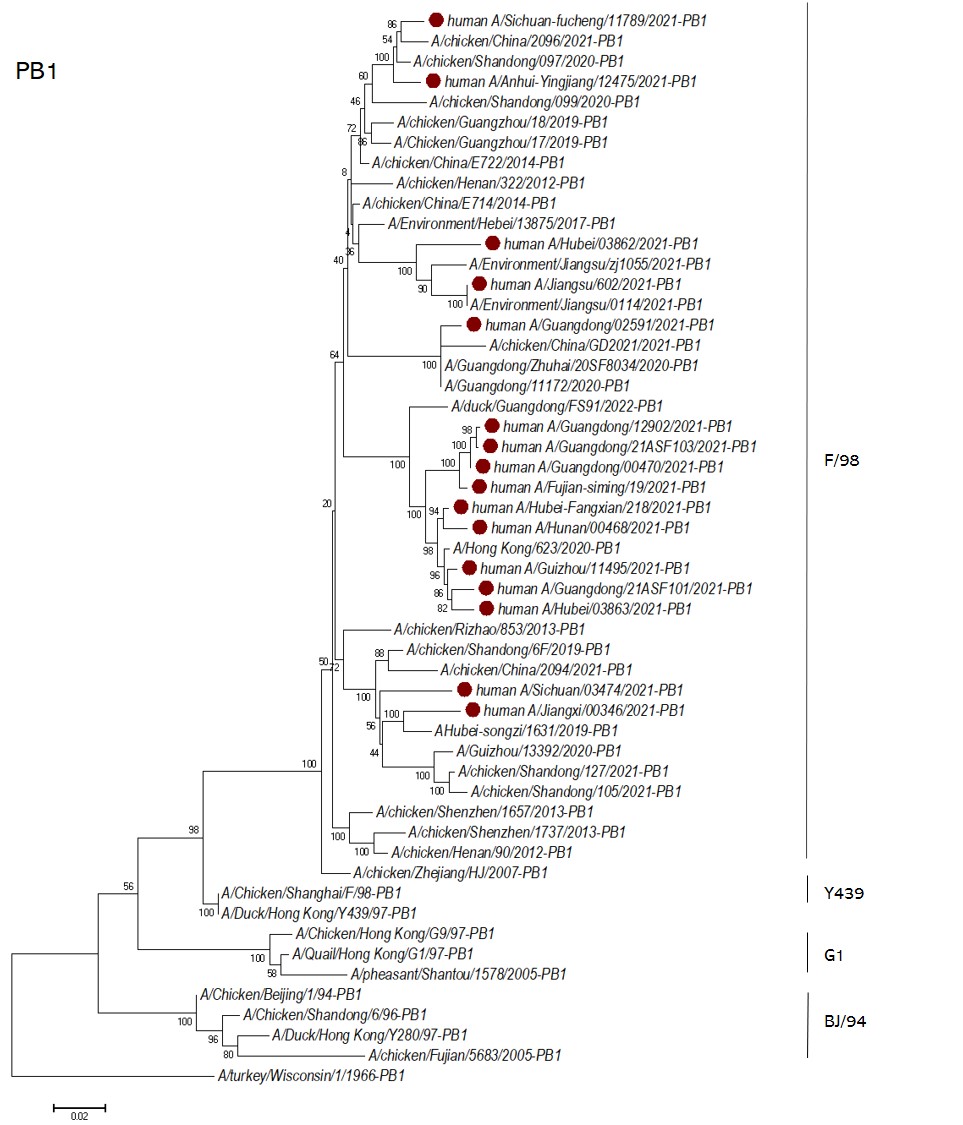

Supplement: Supplementary file 4 [file Image_4.JPEG]

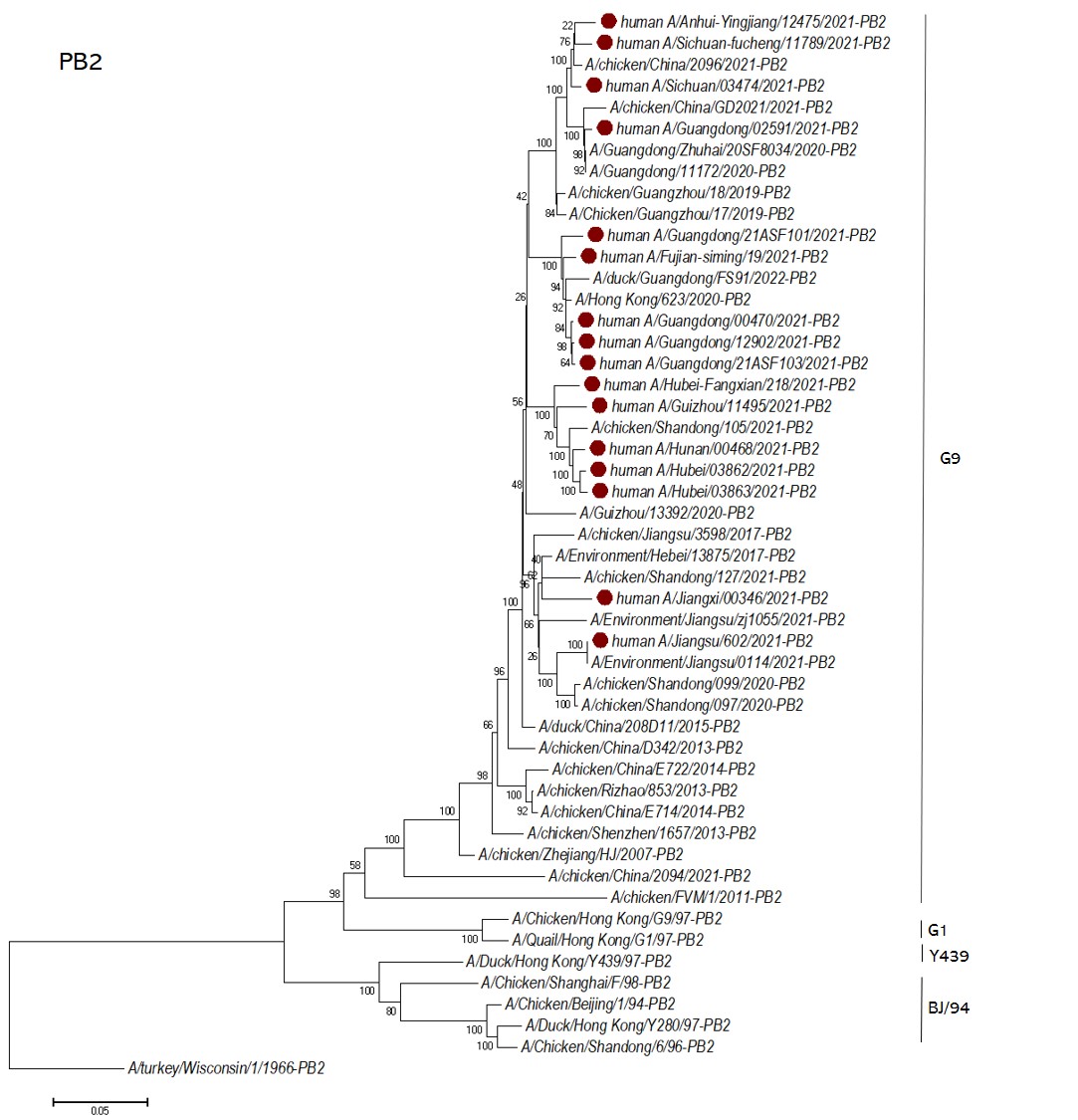

Supplement: Supplementary file 5 [file Image_5.JPEG]

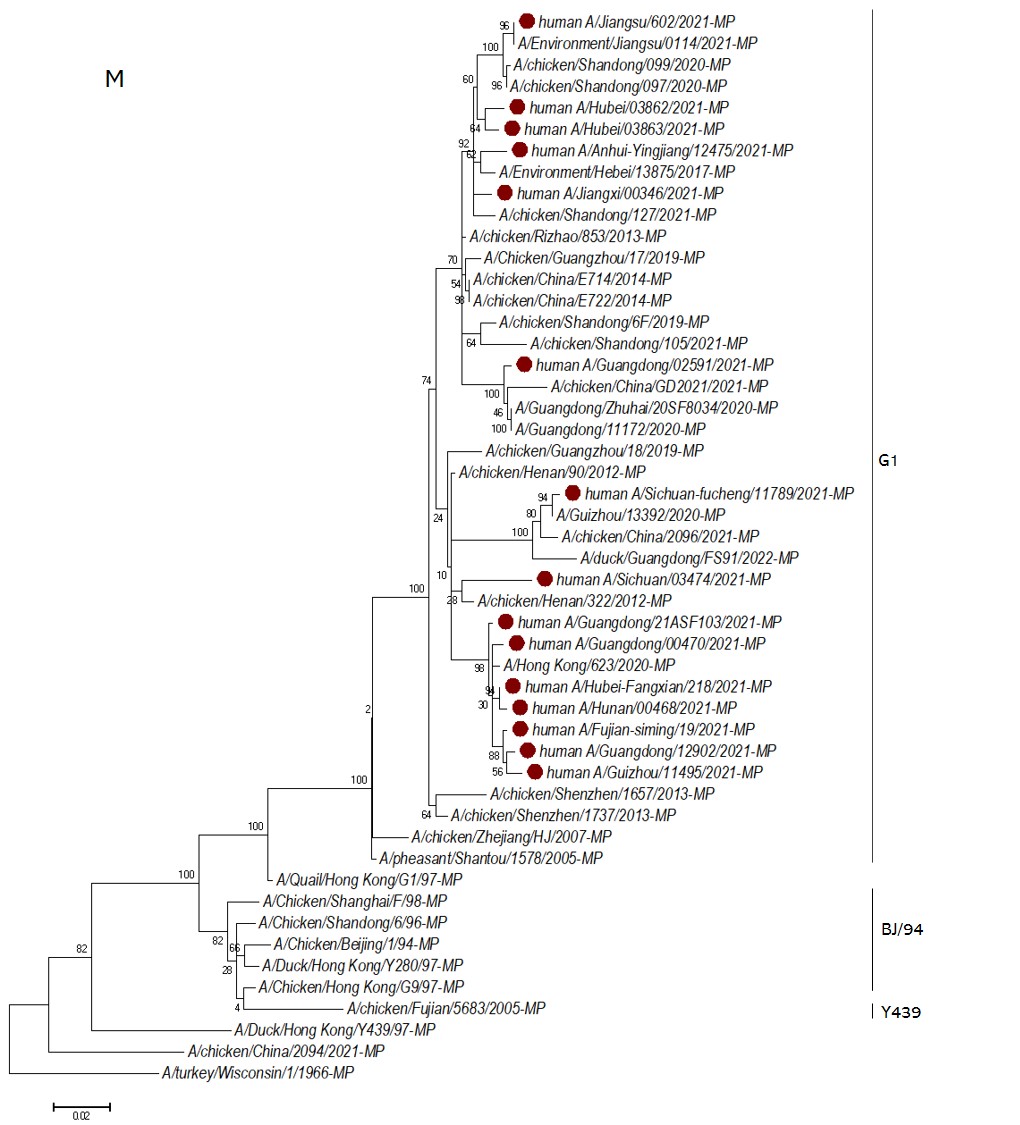

Supplement: Supplementary file 6 [file Image_6.JPEG]

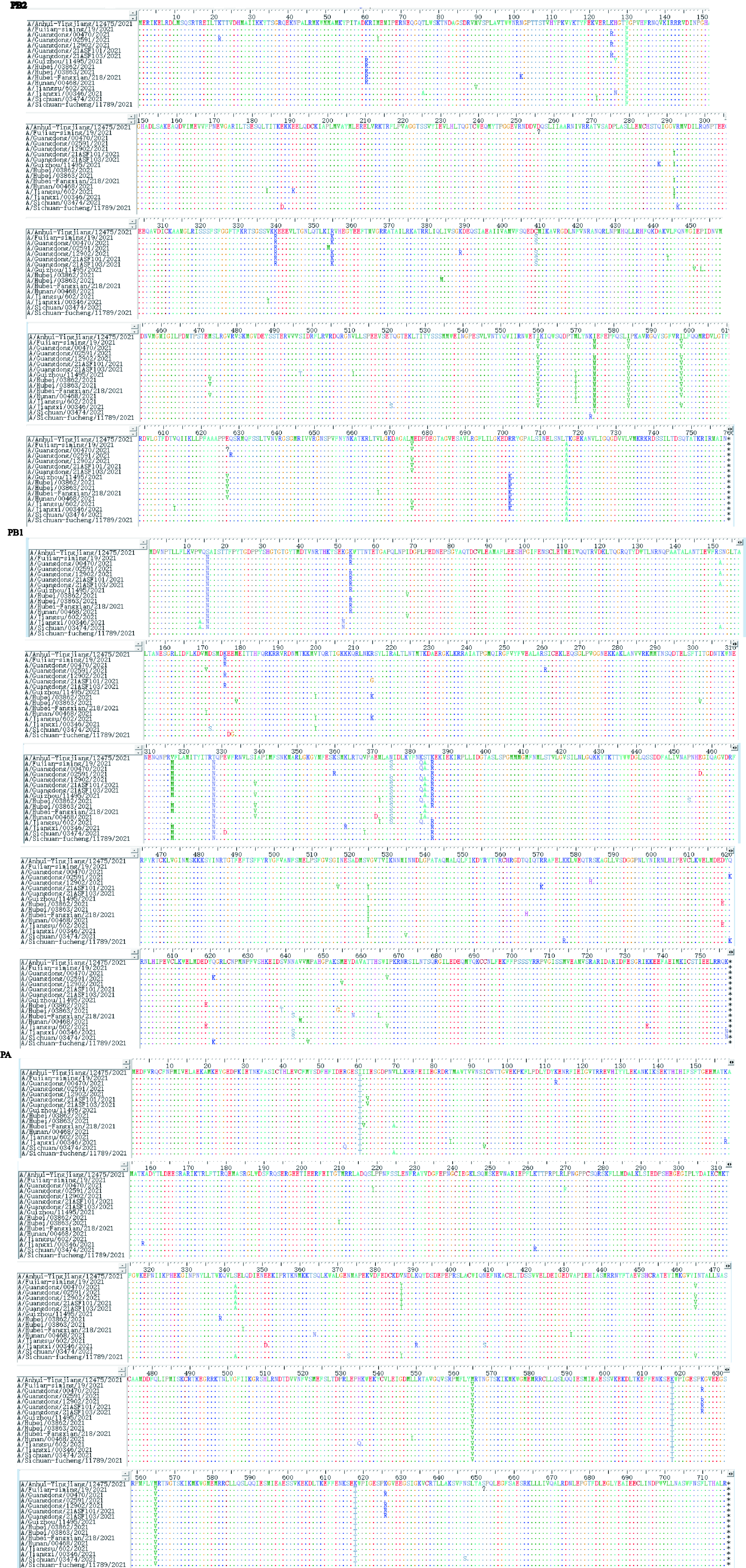

Supplement: Supplementary file 7 [file Image_7.JPEG]

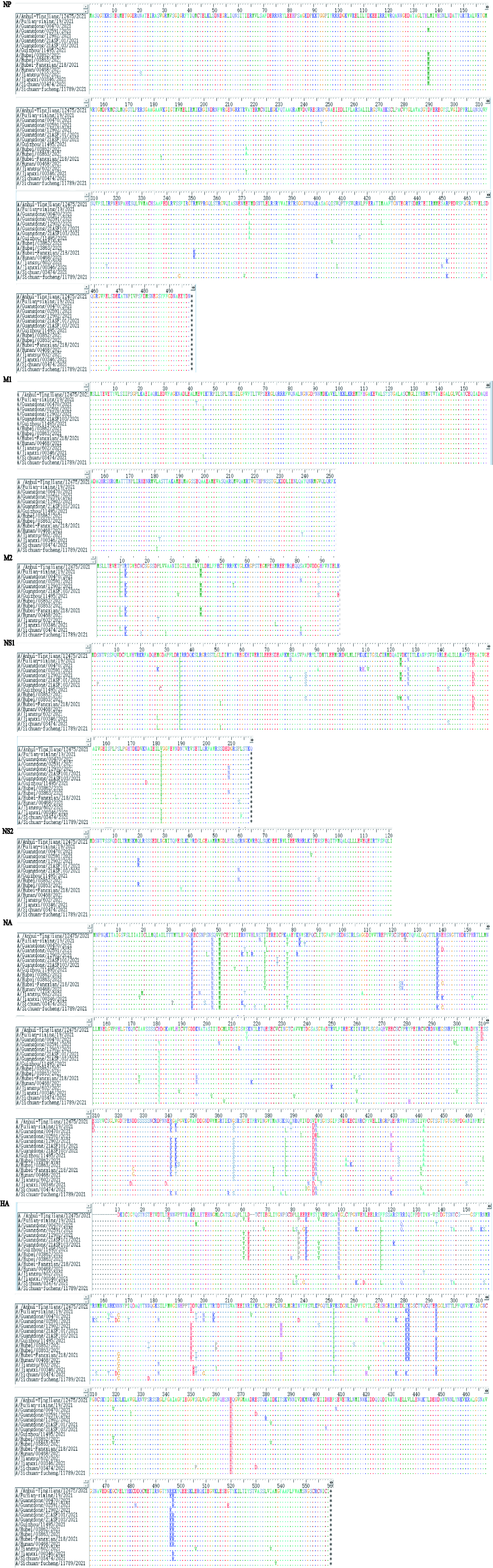

Supplement: Supplementary file 8 [file Image_8.JPEG]
